# For the times they are a-changin’?

**DOI:** 10.1186/s42155-018-0045-x

**Published:** 2019-01-08

**Authors:** Jim A. Reekers

**Affiliations:** Department of Interventional Radiology, Academic Medical Center, 1105 AZ Amsterdam, NL The Netherlands

One of the main characteristics of interventional radiology innovations is that the introduction of new technologies is so rapid that we hardly have enough time to properly evaluate what we are doing. The constant head-over-heels introduction of new endovascular technologies sometimes looks more like a rat race in which a new scoop should be presented at every meeting. Good and long-term evaluation, which assesses all potential risks, is therefore often not possible. Fast introductions and short-term follow-ups do not only contain safety risks, but also hold the risk of losing focus on the clinical goals for the new technologies.

Recently there have been serious doubts expressed by the National Institute for Health and Clinical Excellence (NICE) about the safety, and especially about the clinical validity of the EVAR concept.

At the time of the writing of this editorial the official NICE publication has been postponed, but anyhow, the word is out. Furthermore, there was a recent publication that focussed on the safety of paclitaxel-eluding technologies used during peripheral interventions (Katsanos et al., 2018). This paper by Katsanos et al. shows a significantly higher mortality rate after two and five years for peripheral PAD patients treated with a paclitaxel-related technologies like DES and DEB. The number needed to harm at 5 years is 14 which is a serious issue.

We all still remember the Björk-Shiley valve, where an unexpected mortality due to a construction fault was a reason for the FDA to withdraw the valve from the market. We have to see how this will develop, but for the time being we should take this warning very seriously without “shooting the messenger” by accusing him of data mining and prematurity. Science is not fake news and the mere fact that one cannot explain a certain finding in a study does not mean it is untrue.

Both cases, NICE and paclitaxel, show again that long-term follow-up for new technologies, and especially implants, is mandatory. Therefore, the European Union has introduced new EU MDR/IVDR legislation, which will regulate the introduction of new implants and in-vivo devices on the European market. This legislation will be law in all European countries in 2020. Some of the key points are: better regulation of notified bodies, stronger demand for clinical efficacy before introduction to the market and regulated pre - and post-marketing control.

This will have a major impact on what IR is doing today. From a European patient’s perspective this is all good news, although some may argue that it will delay the introduction of promising new technologies and that patients will not be able to have access to this for a long time. Actually, this was the very argument for the introduction of the FDA 510(k) clearance system, where new technologies are rapidly introduced based on comparisons with related recently introduced technologies, which are often also not well evaluated. It looks a little bit like a Ponzi scheme. After the introduction of this new legislation, clinical efficacy, especially, will be a hot debate.

In endovascular treatment we very often use so-called proxy endpoints to prove efficacy, and our vision has been blurred in such a way that many now confuse proxy endpoints with real clinical endpoints. The great advantage of proxy endpoints is that they are often easy to reach with small study groups. It also offers the opportunity to include study patients with minor disease, as the lesion is studied and not the patient. However, they are also highly subjective or manipulative which makes them a soft target for any study design.

One of the favourite proxy endpoints in endovascular treatment studies is target lesion revascularization (TLR). The target lesion is, for example, a mid-superficial femoral artery stenosis. The TLR investigates the time to re-intervention at the same lesion site. However, the indication for re-intervention is almost always unclear and often physician- or duplex-driven. The clinical condition is hardly ever used as the indication for re-intervention. It also completely denies that the patient is more than a lesion and that atherosclerosis is a often a multi-segmental disease.

The IMPERIAL trial, which was recently published in the Lancet, very clearly shows how TLR has moved the patient completely out of the field of interest of the investigators (Gray et al., 2018). This paper is a competitive, randomised non-inferiority study between 2 paclitaxel eluting stents. The majority of patients included had Rutherford 2–3, which is mild and severe intermittent claudication. By the way, the patients would have been very well off with supervised exercise therapy (SET), an equally effective treatment for claudication due to SFA disease (Mazari et al., 2012). In this study there was a significant improvement in TLR both in the older drug-eluting stent (ZILVERPTX) as in the newer competitor (Eluvia), which is seen as evidence for the efficacy of this technology.

However, all of that aside, let us for a moment focus on the primary goal of our existence as physicians – to help our patients. First of all, the entrance complaint of a patient with intermittent claudication is his/her limitation of walking distance and due to that a decrease in quality of life (QOL). Therefore, our treatment should be aimed at remedying that problem. In summary, treatment for IC is aimed at improving walking distance and QOL. After one year, patients in the IMPERIAL trial had a marginal gain of 50 m walking distance in six minutes, despite a normal ankle-brachial index. However, 50 m is completely irrelevant to any patient, especially those who already walk almost 300 m at baseline (Conijn et al., 2016). Concerning QOL, the EQ-5D scores change from 0.70 ± 0.2 to 0.80 ± 02 after one year in the Eluvia group and from 0.80 ± 0.1 to 0.80 ± 0.2 in the Zilver group, unclear if this is a significant change from baseline. Finally, the aneurysmal degeneration in six patients in the Eluvia group is worrying. However, before I forget – the TLR improved very significantly in both groups.

Are we looking at a house falling apart because the foundation and the concrete holding it together are of inferior quality? Time will tell, but it does not look good.

What can we learn as interventionalists? First of all, we should start to put the patient in the focus of our attention again – not only his lesion. Doctors should be doctors. Secondly, a more rigid post-market surveillance with an early warning system should be installed for all new IR technologies. And finally, the gasping urge to come up with new developments every year, even though the gadgets of last year have yet to be investigated properly, should stop. It is not too late yet. Come, doctors and scientists who prophesise with your pen, and keep your eyes wide – the chance won’t come again!
